# SARS-CoV2 drives JAK1/2-dependent local and systemic complement hyperactivation

**DOI:** 10.21203/rs.3.rs-33390/v1

**Published:** 2020-06-09

**Authors:** Bingyu Yan, Tilo Freiwald, Daniel Chauss, Luopin Wang, Erin West, Jack Bibby, Matthew Olson, Shahram Kordasti, Didier Portilla, Arian Laurence, Michail S Lionakis, Claudia Kemper, Behdad Afzali, Majid Kazemian

**Affiliations:** 1 Departments of Biochemistry and Computer Science, Purdue University, West Lafayette, IN, USA; 2 Immunoregulation Section, Kidney Diseases Branch, National Institute of Diabetes and Digestive and Kidney Diseases (NIDDK), NIH, Bethesda, MD, USA; 3 Laboratory of Molecular Immunology and the Immunology Center, National Heart, Lung, and Blood Institute (NHLBI), National Institutes of Health (NIH), Bethesda, MD, USA; 4 Department of Biological Sciences, Purdue University, West Lafayette, IN, USA; 5 School of Immunology and Microbial Sciences, Faculty of Life Sciences and Medicine, King’s College London, London, UK; 6 Division of Nephrology and the Center for Immunity, Inflammation and Regenerative Medicine, University of Virginia, VA, USA; 7 Nuffield Department of Medicine, University of Oxford, UK; 8 Fungal Pathogenesis Section, Laboratory of Clinical Immunology and Microbiology, National Institute of Allergy and Infectious Diseases (NIAID), NIH, Bethesda, MD, USA; 9 Institute for Systemic Inflammation Research, University of Lübeck, Lübeck, Germany

**Keywords:** Complement, C3, COVID-19, SARS-CoV2, Ruxolitinib, STAT1, RELA, RNA-sequencing

## Abstract

Patients with coronavirus disease 2019 (COVID-19) present with a range of devastating acute clinical manifestations affecting the lungs, liver, kidneys and gut. The best-characterized entry receptor for the disease-causing virus SARS-CoV2, angiotensin converting enzyme (ACE) 2, is highly expressed in these tissues. However, the pathways that underlie the disease are still poorly understood. Here we show that the complement system is unexpectedly one of the intracellular pathways most highly induced by SARS-CoV2 infection in lung epithelial and liver cells. Within cells of the bronchoalveolar lavage of patients, distinct signatures of complement activation in myeloid, lymphoid and epithelial cells tracked with disease severity. Modelling the regulome of host genes induced by COVID-19 and the drugs that could normalize these genes both implicated the JAK1/2-STAT1 signaling system downstream of type I interferon receptors, and NF-κB. Ruxolitinib, a JAK1/2 inhibitor and the top predicted pharmaceutical candidate, normalized interferon signature genes, IL-6 (the best characterized severity marker in COVID-19) and all complement genes induced by SARS-CoV2, but did not affect NF-κB-regulated genes. We predict that combination therapy with JAK inhibitors and other agents with the potential to normalize NF-κB-signaling, such as anti-viral agents, may serve as an effective clinical strategy.

Coronavirus disease 2019 (COVID)-19, a novel viral pneumonia caused by a new beta coronavirus named severe acute respiratory syndrome coronavirus (SARS-CoV)-2, is now a global pandemic. Patients with COVID-19 present with a variable clinical syndrome, ranging from a mild coryzal illness in the majority to a significant minority that develop severe and life-threatening complications, characterized by combinations of acute respiratory distress syndrome, coagulopathy, vasculitis, kidney, liver and gastrointestinal injury (1). Survivors, and those with milder presentations, may suffer from loss of normal tissue function due to persistent inflammation and/or fibrosis. This may lead to the development of chronic respiratory (including infections and chronic airways disease), cardiovascular and renal diseases. The pathogenesis of COVID-19 and its variable severity is currently poorly understood. A better mechanistic understanding of the disease will help identify at-risk patents, develop and refine much-needed pharmaceutical strategies for treatment of COVID-19 and preservation of healthy tissues.

To gain insights into the pathophysiologic mechanisms of COVID-19, we sourced bulk RNA-seq data from lung tissues of two patients with SARS-CoV2 infection and healthy controls (Table S1A) (2). We compared the transcriptomes of patients to controls using gene enrichment analysis (GSEA) (3) and found 36 canonical pathways curated by the Molecular Signatures Database (MSigDB) to be induced in patients compared to controls (Fig. 1A and Table S1B). Our interest was drawn to complement, because five of the 36 (14%) enriched pathways were annotated as complement pathways. The complement system is an evolutionarily conserved component of innate immunity, required for pathogen recognition and removal. The key components are complement (C)3 and C5, which circulate in their pro-enzyme forms in blood and interstitial fluids. C3 is activated through the classical (antibody signal), lectin (pattern recognition signal) and/or alternative (altered-self and tick-over) pathways into C3a and C3b. C3b generation triggers subsequent activation of C5 into C5a and C5b, with the latter seeding the formation of the lytic membrane attack complex on pathogens or target cells. C3a and C5a are anaphylatoxins and induce a general inflammatory reaction by binding to their respective receptors, C3a receptor (C3aR) and C5aR1 expressed on immune cell. Traditionally, complement is considered a mostly hepatocyte-derived and serum-effective system. Thus, the dominance of the SARS-CoV2-induced lung cell-intrinsic complement signature was unexpected. Since the patient lung biopsy samples contained a mixed population of lung cells, we next aimed at defining the cellular source of complement in the affected lungs. To this end, we examined the transcriptomes of primary human bronchial epithelial (NHBE) cells infected *in vitro* with SARS-CoV2, which again identified several complement pathways as highly enriched in infected cells. Hierarchical classification of enriched pathways by significance (FDR q-value) showed that complement pathways were among the most highly enriched of all pathways following SARS-CoV2 infection (Fig. 1B). SARS-CoV2 particularly infects type II pneumocytes, which are the highest expressers of ACE2, the best characterized entry receptor for the virus (4). We, therefore, examined the transcriptomes of type II human pneumocyte cell line, A549, infected with SARS-CoV2, as well as A549 cells first transduced to express high levels of ACE2. Complement pathways were among the most highly enriched, one of which was the most significantly induced pathway in ACE2-transduced A549 cells (Fig. 1C) (2). This response appeared somewhat specific for SARS-CoV2 as analysis of RNA-seq of influenza A-infected HNBE or influenza A- or RSV-infected A549 cells did not induce such dramatic enrichment (Figs. 1B-C and Table S1B).

To further pinpoint common modes of function, we compared all SARS-CoV2-induced pathways among the four sample types infected with this virus: patient lung biopsies, NHBE, A549 and A549-ACE2 cells. There were 14 pathways that were common to all sample types, four of which, approximately ~30%, were complement dependent (Figs. 1D-E). The other shared pathways included predominantly antiviral responses, especially type I interferons (IFNs) (Fig. 1E). Taking the KEGG complement and coagulation pathway as an exemplar, we noted that genes whose transcription was most highly induced by SARS-CoV2 were encoding components of the C1 proteases C1R and C1S, complement factor B (CFB) and complement C3 (Figs. 1F-H and Fig. S1A). This was supported by apparent dose-dependency between SARS-CoV2 viral loads in infected samples and C3 expression (Fig. 1I). To further test our conclusions, we analyzed transcriptomes of cells obtained from nasal washes of ferrets infected with SARS-CoV2, a model of COVID-19. This also showed local induction of *C3, CFB, C1S* and *C1R* gene transcription between 7-14 days post-infection, providing corroborating *in vivo* evidence (Fig. S1B and Table S1C). C1 proteases are initiators of the classical pathway of complement activation, CFB is essential for the alternative pathway of complement activation and C3 is the fundamental rate-limiting substrate for both. Collectively, these data provide evidence that the complement system is one of the top pathways activated by SARS-CoV2 in lung epithelial cells and suggest that respiratory epithelial cells are a primary source of complement within lung tissues, where serum-derived complement is absent. One clinical study reported the presence of both proximal (C4d) and distal complement fragments (C5b-9) in lung tissues (5), corroborating sustained local activation of this system. Data from ferrets indicating heightened transcription of these factors between 7- and 14-days post-infection is consistent with the approximate timeline of clinical decline in the majority of patients with severe COVID-19. Of note, a number of coagulation cascade components, including fibrinogen alpha, beta and gamma chains (FGA, FGB and FGG) were also induced by SARS-CoV2 in respiratory epithelial cells, suggesting that local provision of these proteins may play a part in the microvascular thromboembolic evident in the lungs of patients with COVID-19 (6) (Figs. 1F-H).

Patients with COVID-19 can have either low (indicating activation and consumption) or normal serum levels of complement (7). The liver is the major source of serum complement. Although direct isolation of SARS-CoV-2 from liver cells has yet to be shown, ACE2 is abundantly expressed in liver cells and a significant incidence of acute liver injury has been reported to occur during the course of the disease among patients with COVID-19 (8). Both SARS-CoV and MERS-CoV, two closely related coronaviruses, have a high incidence of liver injury and directly infect liver cells (9, 10), suggesting that the same is true of SARS-CoV2. We therefore assessed whether the liver might also alter complement output upon infection. We compared the transcriptomes of the human hepatocyte cell line, Huh7, infected with SARS-CoV2 (Table S1D) against uninfected cells using GSEA. At 24h post-infection we saw little evidence for the induction of complement components. By contrast, complement was the most significantly induced pathway after 48h (Fig. S1C and Table S1E). As before, we focused on the KEGG complement and coagulation pathway and found that the majority of all complement genes were significantly induced by SARS-CoV2 in liver cells (Figs. S1D-F). Such SARS-CoV2-driven complement hyper-production by the liver could potentially account for the puzzling observation that patients with COVID-19 often display normal serum complement levels despite evidence for profound systemic activation of complement. Transcription of a large number of coagulation cascade genes were induced by SARS-CoV2 in liver cells (Fig. S1E), which may contribute to the profound coagulopathy seen in severe COVID-19 (1, 6, 11).

To obtain insight into the interactions between SARS-CoV2 and the complement system *in vivo*, we analyzed the only publicly available single cell RNA-sequencing data from patients with COVID-19 (12), as such data are highly relevant in understanding host-virus interactions (13). Bronchoalveolar lavage (BAL) samples from patients with mild (n=3) and severe (n=3) COVID-19 were compared with lung biopsy samples from healthy individuals (n=8). Clustering across all cells revealed three major cell types of myeloid, lymphoid and epithelial origin, with seven apparent sub-cell types. We distinguished Type I (AT1) and Type II pneumocytes (AT2) (Figs. 2A-B). We found that expression of C3 was highest in AT2 cells (Fig. 2C, **left panel** and **top panels** of Figs. S2A-B), which correspondingly have the highest expression of ACE2, the entry receptor for SARS-CoV2 and have been identified as the major targets of primary SARS-CoV2 infection (4) [note that absolute cellularity is different between patients and healthy subjects due to differences in tissue source; lung biopsy versus BAL, respectively]. Expression of C3 was significantly higher in AT2 cells of patients with COVID-19 than those of healthy donors, indicating that *in vivo* coronavirus infection induces *C3* gene transcription.

C3 is cleaved into two principal biologically-active components, C3a and C3b, which bind their cognate receptors C3aR and CD46, respectively, on leukocyte subsets. *C3AR1*, the gene encoding C3aR protein, was mainly expressed on myeloid cells (Fig. 2C **middle panel** and **middle panels** of Figs. S2A-B) and CD46 was highly expressed on lymphoid cells (Fig 2C **right panel** and **lower panels** of Figs. S2A-B). To determine whether C3 within lung tissues is biologically active, we looked for the signature of genes regulated by its cognate activation fragment receptors. We curated a list of CD46 target genes in lymphoid cells and a list of C3aR targets in myeloid cells and sourced IFN-α/β signaling genes (Reactome: R-HSA-909733) (Table S2). Expression of CD46-regulated genes was significantly higher in lymphoid cells of patients and was higher in more severe cases (Figs. 2D-E top panels). Similarly, C3aR-regulated gene expression was significantly higher in myeloid cells of patients compared to those from healthy individuals (Figs. 2D-E **middle panels**). Collectively, these data indicate that SARS-Cov2 infection drives *C3* gene transcription locally in lung epithelial cells. Further, SARS-CoV2-induced C3 leads to local biologically active C3 products that invoke distinct complement signatures across lymphoid, myeloid and epithelial cells in patients at the single cell level. Because these gene signatures are inflammatory, our interpretation is that C3 ligation of its receptors on tissue resident/infiltrating leukocytes in COVID-19 is pathogenic. This is supported by a mouse model of SARS-CoV infection, a related virus of the same family, in which C3, C1r and Cfb are all part of a pathogenic gene signature correlating with lethality (14), and in which global *C3^−/−^* status is protective (15). The use of the C3 inhibitor AMY-101 in one patient with COVID-19, who recovered (16), and the C5 activation inhibitor, eculizumab, as adjunctive treatment in four patients, who recovered (17), provide clinical evidence that complement may play a pathogenic role in COVID-19. With few exceptions, complement activity is usually protective during viral infections and critically required to control the pathogen (18). The mechanism that converts this usually protective system into a harmful one during COVID-19 is currently unclear – but may be rooted in the overwhelming combined local and systemic complement induced by the virus.

We next evaluated the extent of type I IFN responses, as IFNs were a common pathway activated by SARS-CoV2 in respiratory epithelial cells (Fig. 1E). These were elevated in patient AT1 and AT2 cells compared to healthy cells (Figs. 2D-E **lower panels**). CD46, C3aR and IFN-α/β signaling genes appeared to closely track disease severity in lymphoid, myeloid and pneumocyte (AT1 and AT2) cells, respectively. The association between both IFN and C3 led us to consider the possibility that there may be a causal relationship between the two.

To gain insights into the regulatory mechanisms underlying the local induction of complement in respiratory epithelial cells and predict potential therapeutic strategies, we used two parallel approaches to identify druggable pathways: transcription factor (TF) prediction and GSEA-based pharmaceutical prediction. In the first, we assessed the genes differentially regulated by SARS-CoV2 in primary normal human bronchial epithelial cells and the type II pneumocyte (A549) cell line. SARS-CoV2 induced 223 and 108, and repressed 178 and 40 genes, in NHBE and A549 cells, respectively (Fig. 3A and Table S3A). We used ingenuity pathway analysis (IPA) to predict the transcriptional regulators of these genes. Of the top ten TFs predicted, half were IFN-pathway signaling proteins, including STAT1, the JAK-1/2-induced STAT that transduces signals downstream of the IFN-α receptor (19). Two of the other core TFs were NF-κB family TFs, including RELA (Fig. 3B), a major regulator of gene transcription in response to pathogen and inflammatory cytokines (e.g. TNF and IL-1β) (20). To explore these observations, we analyzed publicly available STAT1 and histone 3 lysine 27 acetylation (H3K27Ac, a marker of active and open chromatin regions) ChIP-Seq datasets curated by ENCODE, as well as a RELA ChIP-Seq dataset from GSE132018. Genes regulated by SARS-CoV2 showed significant enrichment for both STAT1 and RELA binding (Fig. 3C). Both TFs bound open chromatin regions (H3K27 acetylated) of genes induced by SARS-CoV2 in COVID-19 patient lung tissue and in normal human bronchial epithelial cells (NHBE) (Fig. S3A-B and Table S3B). This indicated that one or both STAT1 and RELA were highly likely to be directly regulating these genes. In particular, we noted strong binding of both STAT1 and RELA at the promoter regions of *C3, CFB, C1S, C1R, IRF9, IRF7* and *IL6* (Fig. 3D and S3C). Together, these data provided strong evidence that complement components involved in both classical and alternative complement activation, as well as IL-6, are regulated by STAT1 and RELA. IL-6 is a cytokine associated with disease severity in COVID-19 patients, and for which clinical trials targeting the IL-6 receptor with tocilizumab are underway. Little is known about the regulation of local complement expression induction; thus, these data have relevance beyond SARS-CoV2 infection.

In parallel, we carried out pharmaceutical drug prediction. We predicted that drugs able to repress the genes induced by SARS-CoV2 might have potential clinical application. We therefore compared the targets of 1657 curated drugs in the drug signatures database (DSigDB) (21) to the genes induced by SARS-CoV2 infection. In both primary NHBE and A549 cells Ruxolitinib, a Janus kinase (JAK)1/2 inhibitor (JAKi) that blocks STAT1 signaling (22), was predicted to be the top candidate for normalization of the SARS-CoV2 gene signature (Figs. 4A-B and Table S4A), consistent with the enrichment of STAT1 binding in genes regulated by this virus (Figs. 3C-D). In both cell types, Baricitinib, a related JAK1/2 inhibitor that can also potentially block viral entry via endocytosis and that has been piloted in a small number of patients with COVID-19 (23), was predicted to be a regulator of the SARS-CoV2-induced transcriptome, but enrichment for this drug was not as dramatic (Fig. 4A). Of interest, atovaquone, an FDA-approved oral drug with a favorable safety profile that is used for the treatment of AIDS-associated opportunistic infections (*Toxoplasma, Pneumocystis*) appeared close to the top of predicted drugs to “normalize” the SARS-CoV2-induced transcriptional changes in A549 cells (Fig. 4A).

As proof of principle, we analyzed the effects of Ruxolitinib on the SARS-CoV2-induced transcriptome by comparing RNA-seq from A549 cells transduced to express ACE2, then infected with SARS-CoV2 in the presence of Ruxolitinib or carrier. We observed three major categories of response: genes induced by SARS-CoV2 that were not normalized by Ruxolitinib (n=135), genes repressed by SARS-CoV2 that were not normalized by Ruxolitinib (n=91) and genes induced by SARS-CoV2 and almost completely normalized by Ruxolitinib (n=87). In this third category were *IL6, IRF7* and *IRF9* and all of the complement components we had previously observed, namely *C1R, C1S, CFB* and *C3* (Fig. 4C and Table S4B). Because JAKi can have off-target effects and because STAT3 can theoretically be activated by the IL-6 produced in response to SARS-CoV2, we sought to attribute complement regulation directly to STAT1. We analyzed publicly available transcriptomes of STAT1 wild-type (*STAT1^+/+^*) and STAT1 knockout (*STAT1^−/−^*) HepG2 liver cells treated, or not, with IFN-α, from GSE98372 (24) (Table S4C). IFN-α only induced the previously identified components of the complement system, *C3, C1R, C1S, CFB*, nor *IRF7* and *IRF9*, in the presence of intact STAT1 (Fig. S4A). Moreover, most SARS-CoV2 induced genes normalized by Ruxolitinib were down-regulated in STAT1^−/−^ cells and did not respond to IFN-α treatment (Fig. S4B). These data indicated that STAT1 is indispensable for inducing these genes. In addition, IL-6 was also not induced by IFN-α treatment in these cells, irrespective of STAT1 status (Fig. S4A), so we concluded that STAT1, not STAT3, is the dominant driver of complement gene regulation. To address the concern that JAK-STAT inhibition impairs anti-viral immunity and de-represses viral replication (25), we quantified SARS-CoV2 viral loads in these samples by aligning raw reads to the viral genome. Ruxolitinib-treatment did not alter viral loads in any of these samples (Fig. S4C). We used Metascape (26) to functionally annotate the genes in these three categories and gain some insights into why all of the genes were not normalized. While genes whose transcription could be normalized by JAK-STAT inhibition were categorized as complement pathway and IFN-regulated, those that were not normalized by Ruxolitinib especially pertained to NF-κB signaling, including toll-like receptor and TNF signaling (both activators of NF-κB), as well as apoptosis and mitosis (Fig. 4D). Taken together, these data indicated that IFN-induced STAT1 is the dominant regulator of local complement production from respiratory epithelial and liver cells and that the JAKi Ruxolitinib neutralizes SARS-CoV2-mediated complement activation but does not fully normalize the transcriptome. Of note, potential connections between complement activity and type I IFN responses during pathogen encounter is currently an unexplored field aside from a study that used full C3-deficient animals and noted suppressive effect of C3 on IFN after exposure of animal to plant virus-like nanoparticles (27). Our data here suggest that the use of a JAKi to normalize all of the proximal components induced by SARS-CoV2 at the gene transcriptional level could represent a more refined approach than targeting a single complement component (e.g. C3 or C5a) with inhibitors that only work in the extracellular space – as we have shown previously, complement activation also occurs in the intracellular space, where it performs functions critical to mounting an effective inflammatory immune response (28, 29) (Fig. 4E). Moreover, interfering with type I IFN signaling can, in some instances redirect immunity to enable control of viral infection (30).

Both NF-κB and STAT1 signaling, as well as complement activity *per se* have been shown to enhance with cellular ageing in other contexts (31-33), which may partially explain why the elderly are at greater risk of hyper-inflammation with COVID-19 than the young. The prediction that the NF-κB pathway is also a regulator of the genes induced by SARS-CoV2 and the failure of Ruxolitinib to normalize a large subset of genes annotated as NF-κB regulated, suggests that monotherapy with Ruxolitinib may be insufficient for the management of this disease. While there are ongoing clinical trials with JAKi for treating patients with COVID-19, our analyses suggest that combining a JAKi, such as Ruxolitinib, with modifiers of NF-κB signaling, for example anti-viral agents (e.g. Remdesivir) or TNF antagonists, may be a better therapeutic approach than monotherapy alone. Because of concerns regarding the use of JAKi in a disease with a propensity to thrombosis, combining a JAKi with a second agent may permit use of lower doses of both drugs, potentially reducing thrombotic adverse effects and reducing risks of viral replication.

## Supplementary Material

Supplement 1

Supplement 2

Supplement 3

Supplement 4

Supplement 5

## Figures and Tables

**Fig. 1.**
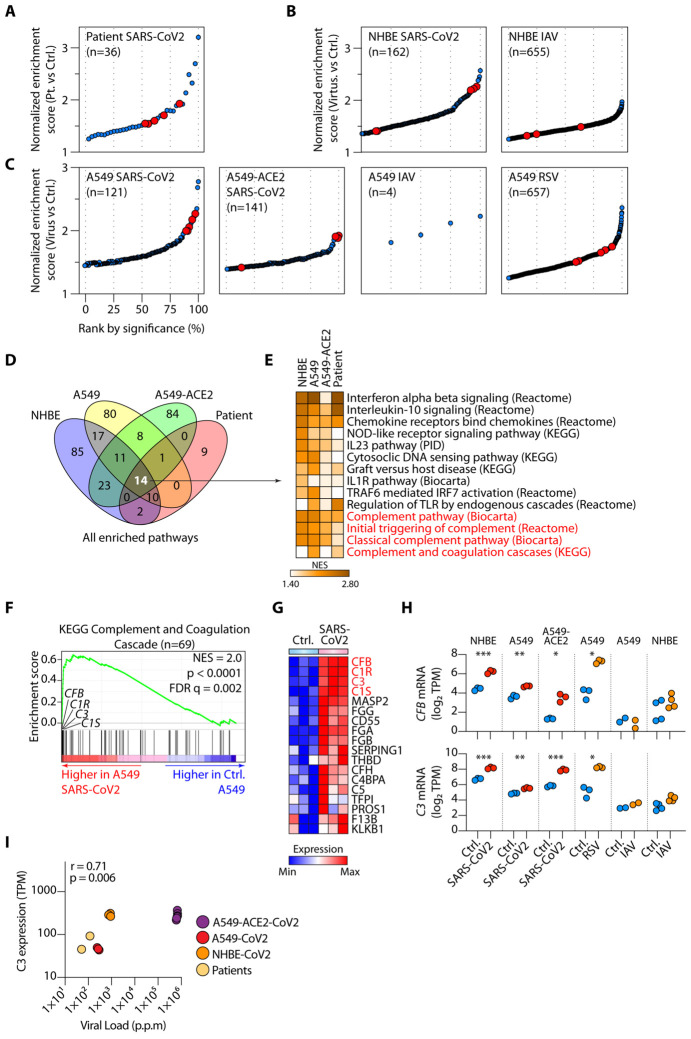
SARS-Cov2 infection activates complement transcription in lung epithelial cells. **(A-B)** Significantly enriched pathways by gene set enrichment analysis (GSEA) comparing transcriptomes of lung samples from SARS-CoV2 infected patients (n=2) versus healthy controls (**A**) and similar GSEA analyses on normal human bronchial epithelial (NHBE) cells infected *in vitro*, or not, with SARS-CoV2 (n=3) (**B**). (**C**) GSEA of human alveolar type II pneumocytes transduced with ACE2 (A549-ACE2) or not (A549), comparing cells infected with SARS-CoV2, Influenza A virus (IAV) or respiratory syncytial virus (RSV) versus control cells (n=3 or 4). In (**A-C**) pathways have been ranked by significance (false-discovery rate FDR q-values), with complement pathways highlighted in red. Only enriched pathways with FDR <0.25 are shown. **(D-E)** Comparison of all pathways significantly induced (FDR q-value < 0.25) by SARS-CoV2 in patients (**A**), NHBE cells (**B**), A549 and A549-ACE2 cells (**C**), indicating 14 shared enriched pathways (**D**) and their normalized enrichment score (NES) displayed as a heatmap, with complement pathways highlighted in red (**E**). (**F-G**) Representative GSEA plot for one of the complement pathways in (**E**) and expression of the leading-edge genes from this pathway, with *C3, C1R, C1S* and *CFB* highlighted in red (**G**). (**H**) Expression of *CFB* (upper panel) and *C3* (lower panel) mRNA in control (Ctrl.) versus SARS-CoV2-infected cells. (**I**) Spearman correlation between *C3* mRNA expression and SARS-CoV2 viral load across virus bearing samples in **Fig 1**. p.p.m: parts per million mapped reads. Data in **Fig. 1** have been sourced from GSE147507. * p <0.05, ** p <0.01, ***p < 0.001 by ANOVA.

**Fig. 2.**
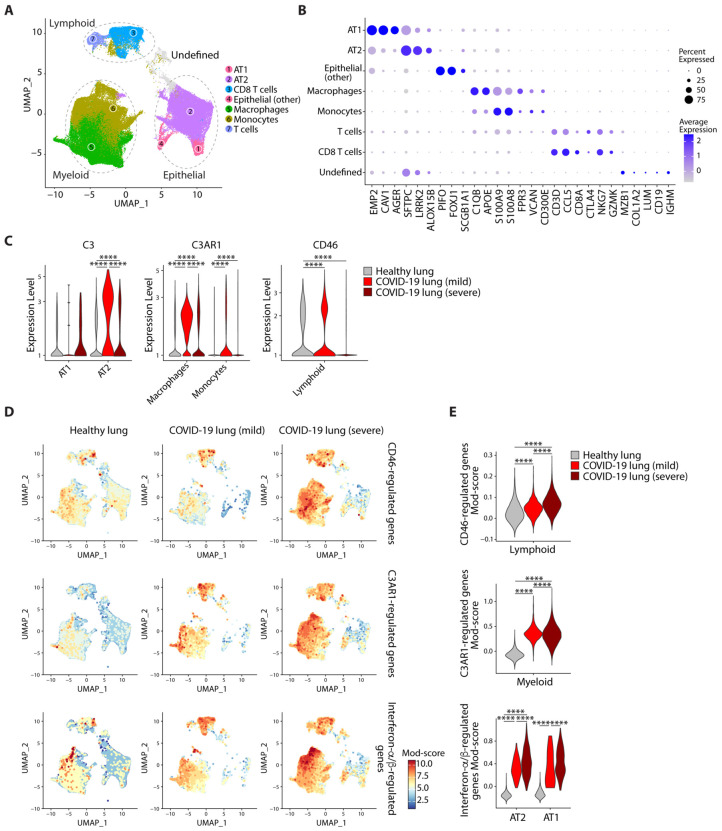
SARS-Cov2 infection invokes distinct complement signatures across lymphoid, myeloid and epithelial cells in patients. **(A)** UMAP showing 3 major cell types and 7 sub-cell types in healthy subject lung biopsies (n=8) and COVID-19 bronchoalveolar lavage (BAL) specimens from patients with mild (n=3) and severe (n=3) COVID-19. (**B**) Expression of cell-defining features across all cell types. (**C**) Expression of *C3, C3AR1* and *CD46* in select cell types across healthy, mild and severe COVID-19 samples (see also Fig. S2A for all cell types). (**D-E**) The UMAP projection (**D**) and module (Mod) score (34) (**E**) of CD46-regulated genes (top panel), C3aR1-regulated genes (middle panel) and interferon-α/β-regulated genes (see Table S2). In (**E**) selected cell types are shown. Single cell data in **Fig. 2** are from GSE145926 and GSE122960.

**Fig. 3.**
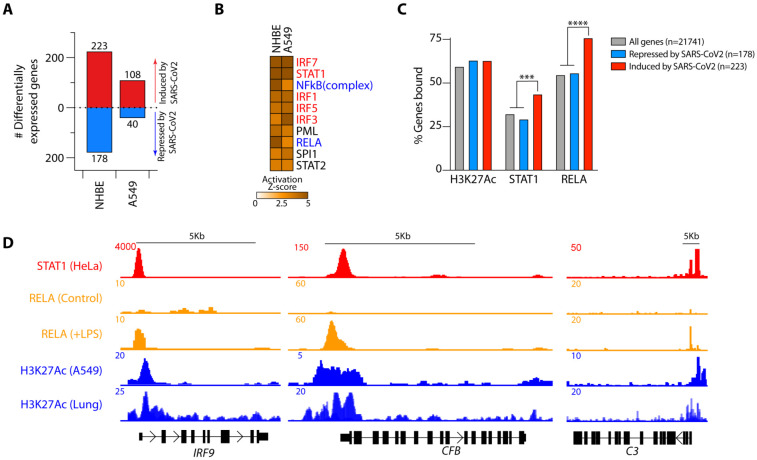
STAT1 and RELA bind Complement genes induced by SARS-CoV2. (**A**) Numbers of differentially expressed genes in normal primary human bronchial epithelial (NHBE) cells and A549 alveolar cell lines infected with SARS-CoV2 in comparison with control virus. (**B**) Top ten Ingenuity Pathway Analysis (IPA) predicted transcription factors (TFs) regulating the SARS-CoV2-driven transcriptional response in normal human bronchial epithelial (NHBE) cells and human alveolar basal epithelial cell lines (A549). Highlighted in red are TFs transducing interferon-mediated and in blue NF-κB-mediated gene transcription. (**C**) Histone 3 lysine 27 acetylation (H3K27Ac) and STAT1 and RELA ChIP-seq binding profiles across SARS-CoV2-induced and repressed genes. (**D**) STAT1, RELA and H3K27Ac ChIP-seq tracks showing the *IRF9, CFB* and *C3* gene loci. Data in **A** are from GSE147507 and in **C-D** have been sourced from ENCODE (H3K27Ac and STAT1) and from GSE132018 (RELA). RELA profiles in **C** are from LPS-treated cells. *** p<0.001; ****p<0.0001 by Fisher’s exact test.

**Fig. 4.**
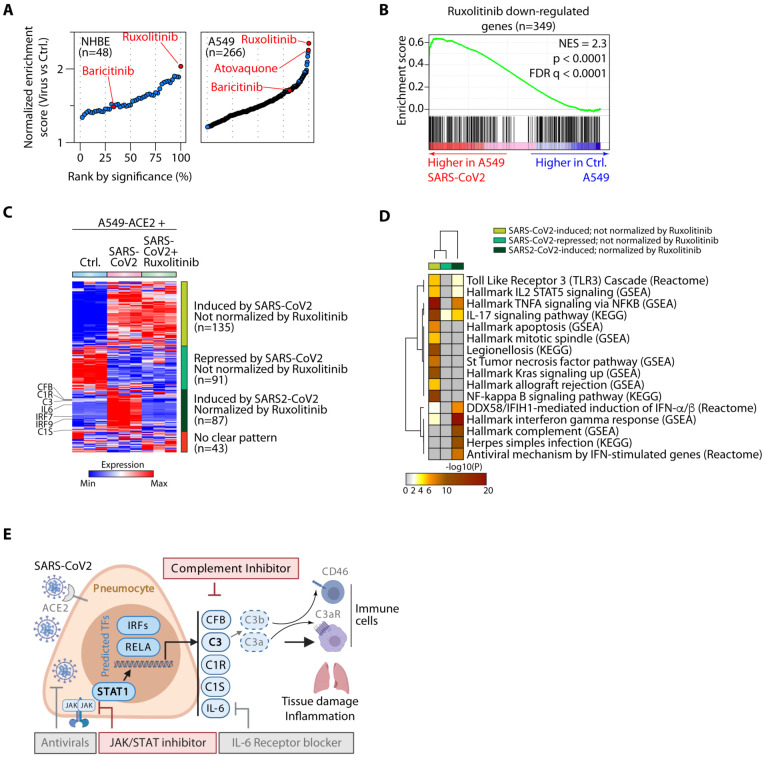
The Janus kinase inhibitor (JAKi) Ruxolitinib neutralizes SARS-CoV2 mediated complement activation. (**A**) Gene set enrichment analysis (GSEA) showing enrichment of genes normalized by pharmaceutical agents in the transcriptomes of control (Ctrl.) or SARS-CoV2-infected NHBE (left) or A549 (right) cells. Drugs have been ranked by significance (false-discovery rate q-values), with Ruxolitinib, Baricitinib and Atovaquone highlighted in red. (**B**) Representative GSEA plot showing enrichment (higher expression) of Ruxolitinib down-regulated genes in SARS-CoV2-treated cells. (**C**) Heatmap showing expression of genes induced/repressed by SARS-CoV2 in A549 cells transduced with ACE2 (A549-ACE2) then infected with SARS-CoV2 in the presence of Ruxolitinib or vehicle. Genes are clustered according to their response to SARS-CoV2 and Ruxolitinib. Highlighted are the patterns of response on the right and selected key complement and interferon pathway genes on the left. (**D**) MetaScape pathway analysis of the three main indicated gene clusters in (**C**). The heatmap shows pathways that are highly significantly enriched (at least one member of each row with p < 10^−5^). (**E**) Schematic showing SARS-CoV2-induced complement gene and *IL6* gene transcription mediated by JAK-STAT signaling downstream of the IFN-α/β receptor and processing of C3 to active fragments that bind receptors on leukocytes that generate tissue inflammation. Inhibitors of different components are illustrated. Schematic created with Biorender.com. Transcriptomes in **Fig. 4** are sourced from GSE147507.
