# Supplementary material for: SARS-CoV2 drives JAK1/2-dependent local and systemic complement hyperactivation
Source: Res Sq. 2020 Jun 9:rs.3.rs-33390. Preprint. [Version 1] doi: 10.21203/rs.3.rs-33390/v1 (PMC7336704; doi:10.21203/rs.3.rs-33390/v1)
Supplement: Supplement 5 [file YanetalComplementandCOVID192020SupplementaryMaterials.pdf]

Supplementary Materials for

SARS-CoV2 drives JAK1/2-dependent local and systemic complement hyper-activation

Bingyu Yan[†], Tilo Freiwald[†], Daniel Chauss[†], Luopin Wang[†], Erin West[†], Jack Bibby, Matthew Olson, Shahram Kordasti, Didier Portilla, Arian Laurence, Michail S Lionakis, Claudia Kemper[*], Behdad Afzali[*], Majid Kazemian[*]

Correspondence to: claudia.kemper@nih.gov; behdad.afzali@nih.gov; kazemian@purdue.edu

This PDF file includes:

Materials and Methods
Figs. S1 to S4

Other Supplementary Materials for this manuscript include the following:

Data Tables S1-S4

Materials and Methods

Gene set enrichment analysis

Gene set enrichment analysis (GSEA) was performed using GSEA version 4.0.3 (*3*) with the following parameters, "Permutation type = gene_set" and "Collapse to gene symbols = No_Collapse". All canonical pathways "c2.cp.v7.1" were used throughout the paper. Upstream transcriptional regulator identification was done using Ingenuity Pathway Analysis (IPA) on genes that were differentially expressed (fold change 1.5, FDR <0.05) in NHBE or A549 cells. Multiple geneset analysis in **Fig. 4** was performed using online MetaScape interface with default parameters on Hallmark Gene Sets (n=44), Canonical Pathways (n=135), Reactome Gene Sets (n=664) and KEGG pathways (n=201).

Bulk RNA-seq data analysis

RNA-seq data was obtained from GSE147507 (Lung samples) and SRP257667 (Liver samples). For GSE147507, the original raw read counts were normalized to obtain transcript per million (TPM) values which were then used for plotting the expression values and performing GSEA analyses. For SRP257667, the raw fastq files were downloaded and mRNA expression levels were estimated by RSEM software (Li and Dewey 2011) using "rsem-calculate-expression" with the following parameters, "--bowtie-n 1 –bowtie-m 100 –seed-length 28". The RSEM required bowtie index was created by "rsem-prepare-reference" on all RefSeq genes downloaded from UCSC table browser on April 2017. The differentially expressed genes were identified using edgeR package (Robinson et al. 2010) on the original raw read counts for GSE147507 and the expected read counts from rsem for SRP257667. Fold change (FC>1.5) and FDR q-value (p<0.05) were used to identify differentially expressed genes.

ChIP-seq data analysis

H3K27Ac ChIP-seq in A549 cells (ENCFF137KNW), H3K27Ac ChIP-seq in primary lung cells (ENCFF055YQO, ENCFF677KZQ) and STAT1 ChIP-seq in HeLa cells (ENCFF000XLN) were obtained from ENCODE. RELA ChIP-seq in FaDu cells was from GSE132018. In all cases, the pre-processed and author-provided peak files (e.g. ENCFF565WST, ENCFF002CTG) were obtained and the nearest transcription start sites (TSS) and corresponding genes were identified by HOMER "annotatePeaks" program. The overlap between these genes and SARS-Cov2 induced/repressed genes or all human genes were then assessed to determine enrichment. ChIP-seq tracks and heatmaps were visualized using IGV browser (Broad Institute) and deepTools (*35*), respectively.

Statistical analysis and data visualization

Analyses were performed using GraphPad PRISM 8 (La Jolla, CA, USA) and Data Graph v4.5. All the individual data points are presented and compared using one-way ANOVA or Fisher exact test, as appropriate. *P* values <0.05 are denoted as statistically significant throughout. The heatmaps were drawn using Morpheus software (Broad Institute).

Drug prediction

Raw read counts for A549 and NHBE cells comparing the SARS-CoV2 infected cells with controls were obtained from GSE147507 and normalized to obtain TPM values (see **Table S1A**). Drugs with provided down-regulated target genes (between 10 to 1000) were obtained from DSigDB v1.0 (*21*). For ruxolitinib, the lists of all up and down-regulated genes (p-value<0.05) were

obtained by comparing MCF-7 cells treated with ruxolitinib or vehicle control (data from GSE131300) using DESeq2 (*36*) (See **Table S4D**). For baricitinib, the list of all up and down-regulated genes (p-value<0.0005) were obtained by comparing systemic baricitinib treatment versus control at 12 weeks (data from GSM1508095) using GEO2R (*37*) (See **Table S4D**). The gene set enrichment analysis (GSEA) was performed using GSEA version 4.0.3 with the following parameters, "Permutation type = gene_set", "Collapse to gene symbols = No_Collapse", "Min Size =10" and "Max Size =1000". A549 and NHBE samples were treated as Expression datasets and the DSigDB data was treated as Gene sets database. All the rest of the parameters were kept as default. The data with FDR q-value <0.25 was reported (See **Table S4A**).

Single Cell RNA sequencing data analysis

The pre-processed h5 matrix files for six COVID-19 patient bronchoalveolar-lavage (BAL) samples and eight healthy control lung biopsies were obtained from GSE145926 and GSE122960, respectively. Read mapping and basic filtering were performed with the Cell Ranger pipeline by the original authors. We further processed the samples using Seurat (version 3) as following: Only genes found to be expressed in more than 3 cells were retained. Cells with >10% of their unique molecular identifiers (UMIs) mapping to mitochondrial genes or cells with <300 features were discarded to eliminate low quality cells or nuclei. This yielded a total of 89133 cells across 14 samples. The filtered count matrices were then normalized by total UMI counts, multiplied by 10,000 and transformed to natural log space. The top 2000 variable features were determined based on the variance stabilizing transformation function (FindVariableFeatures) by Seurat with default parameters. All samples were integrated using canonical correlation analysis (CCA) function with default parameters. Variants arising from library size and percentage of mitochondrial genes were regressed out by the ScaleData function in Seurat. Principal component analysis (PCA) was performed and the top 30 Principal components (PCs) were included in a Uniform Manifold Approximation and Projection (UMAP) dimensionality reduction. Clusters were identified on a shared nearest neighbor (SNN) modularity graph using the top 30 PCs and the original Louvain algorithm. Cluster annotations were based on canonical marker genes. Gene list scores were calculated by AddModuleScore function in Seurat (*34*). Statistical differences of marker expressions and scores were assessed by Wilcoxon test.

Fig S1

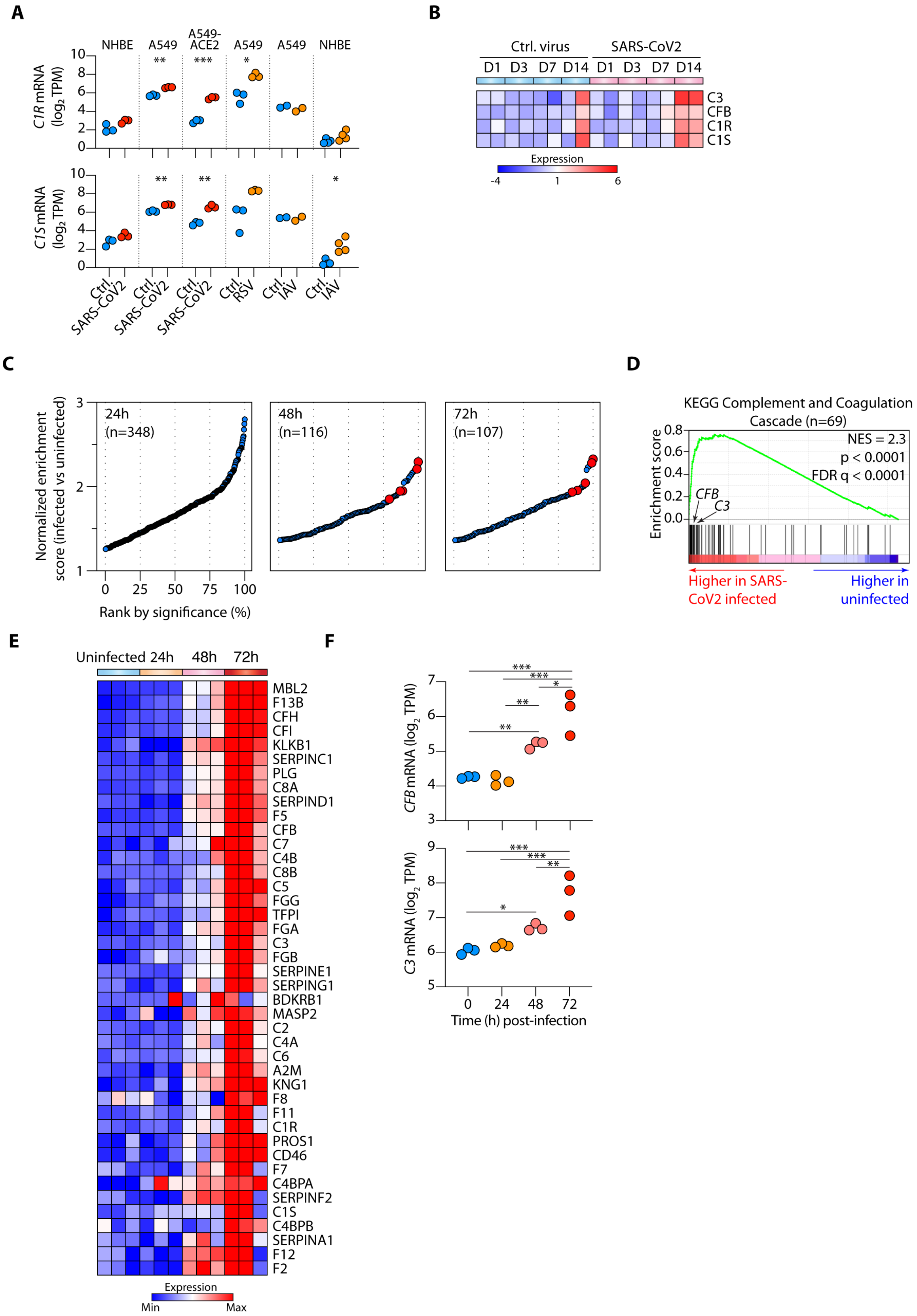
A
NHBE
A549
A549-ACE2
A549
A549
NHBE
**
***
*
C1R mRNA (log2 TPM)
**
**
*
C1S mRNA (log2 TPM)
10
8
6
4
2
0
Ctrl.
SARS-CoV2
Ctrl.
SARS-CoV2
Ctrl.
SARS-CoV2
Ctrl.
RSV
Ctrl.
IAV
Ctrl.
IAV
B
Ctrl. virus
SARS-CoV2
D1 D3 D7 D14 D1 D3 D7 D14
C3
CFB
C1R
C1S
Expression
-4
1
6
C
24h
(n=348)
48h
(n=116)
72h
(n=107)
Normalized enrichment score (infected vs uninfected)
3
2
1
0
25
50
75
100
Rank by significance (%)
D
KEGG Complement and Coagulation Cascade (n=69)
NES = 2.3
p < 0.0001
FDR q < 0.0001
Enrichment score
0.8
0.6
0.4
0.2
0.0
CFB
C3
Higher in SARS-CoV2 infected
Higher in uninfected
E
Uninfected 24h 48h 72h
MBL2
F13B
CFH
CFI
KLKB1
SERPINC1
PLG
C8A
SERPIND1
F5
CFB
C7
C4B
C8B
C5
FGG
TFPI
FGA
C3
FGB
SERPINE1
SERPING1
BDKRB1
MASP2
C2
C4A
C6
A2M
KNG1
F8
F11
C1R
PROS1
CD46
F7
C4BPA
SERPINF2
C1S
C4BPB
SERPINA1
F12
F2
Expression
Min
Max
F
***
***
*
**
**
CFB mRNA (log2 TPM)
7
6
5
4
3
***
***
**
*
C3 mRNA (log2 TPM)
9
8
7
6
5
0
24
48
72
Time (h) post-infection

Fig. S1. SARS-Cov2 infection activates complement transcription in lung epithelial cells, *in vivo* SARS-CoV2 infected ferrets and human liver cells. (**A**) Expression of *C1R* (upper panel) and *C1S* (lower panel) mRNA in control (Ctrl.) versus SARS-CoV2-infected cells in the indicated samples. (**B**) Heatmap showing expressions of *C3*, *CFB*, *C1R* and *C1S* over time in nasal washes of ferrets infected with SARS-CoV2 or sham-infected. **(C)** Significantly enriched pathways by gene set enrichment analysis (GSEA) comparing transcriptomes of human hepatocyte cell lines (Huh7) infected, or not, with SARS-CoV2 (n=3) at 24, 48 and 72hr post infection. Pathways have been ranked by significance (false-discovery rate q-values), with complement pathways highlighted in red. Only enriched pathways with FDR <0.25 are shown. (**D-E**) Representative GSEA plot for one of the complement pathways in (**C**) at 72hr post infection (**D**) and heatmap showing the expression of the leading-edge complement genes across all time points (**E**). (**F**) Expression of *CFB* (upper panel) and *C3* (lower panel) mRNA in control (0hr) versus infected Huh7 cells at all time points. Data have been sourced from GSE147507 (**A-B**) and SRP257667 (**C-F**). * $p < 0.05$, ** $p < 0.01$, ***$p < 0.001$ by ANOVA.

Fig S2

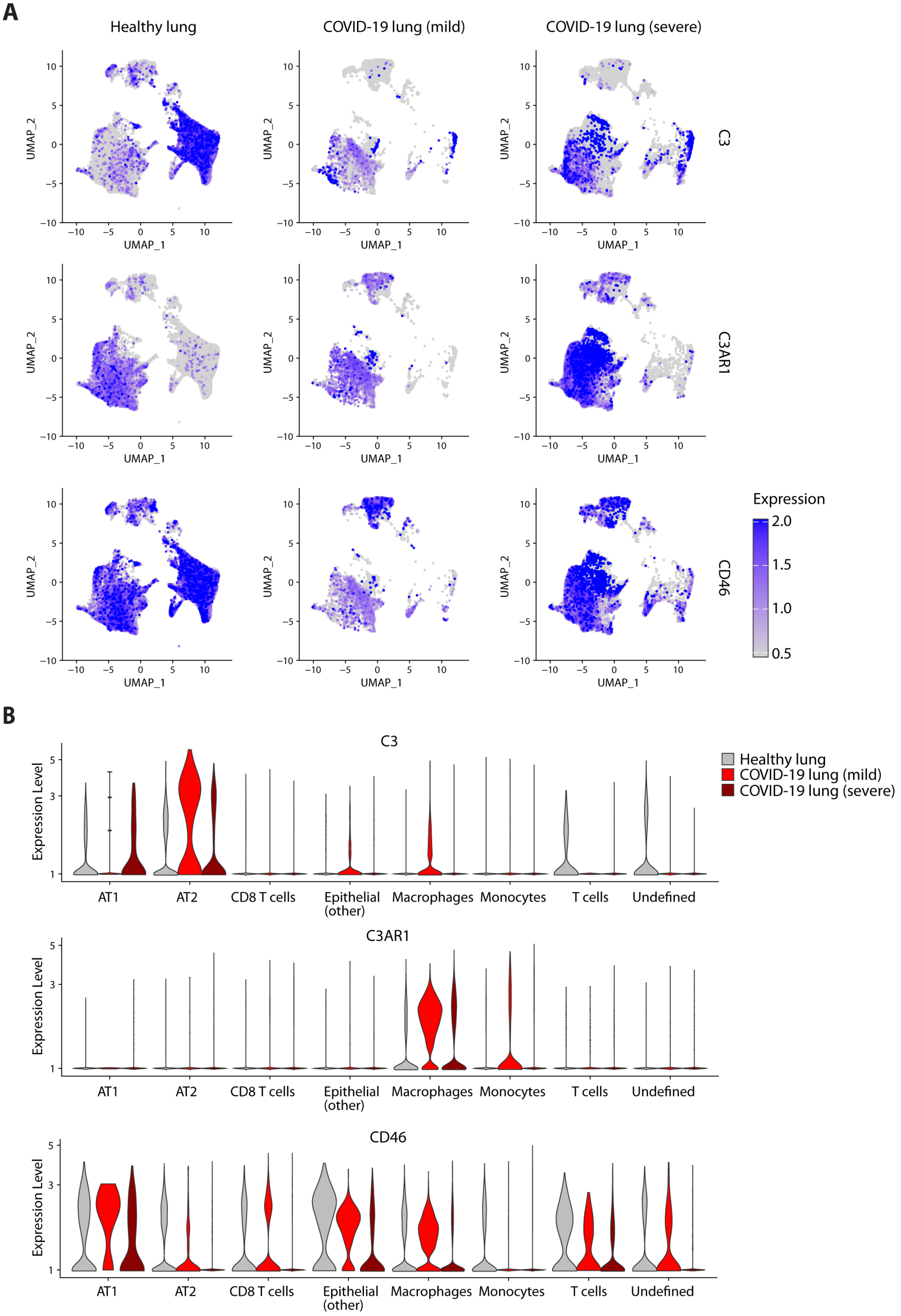
A
Healthy lung
COVID-19 lung (mild)
COVID-19 lung (severe)
C3
C3AR1
CD46
UMAP_1
UMAP_2
Expression
2.0
1.5
1.0
0.5
B
C3
C3AR1
CD46
Expression Level
Healthy lung
COVID-19 lung (mild)
COVID-19 lung (severe)
AT1
AT2
CD8 T cells
Epithelial (other)
Macrophages
Monocytes
T cells
Undefined

Fig. S2. Complement components are expressed in lymphoid, myeloid and epithelial cells. (**A**) the UMAP projection showing C3, C3AR1 and CD46 expression across healthy, mild and severe COVID-19 samples. (**B**) Cumulative data of CD46-regulated genes (top panel), C3aR-regulated genes (middle panel) and interferon α/β regulated genes (see **Table S2**) in all major cell types. Please note that healthy donor samples are from lung biopsies whereas patient samples are from bronchoalveolar lavage, therefore the absolute cellularities are different and the projection of features on the UMAP in (**A**) appears denser for healthy donor cells.

Fig S3

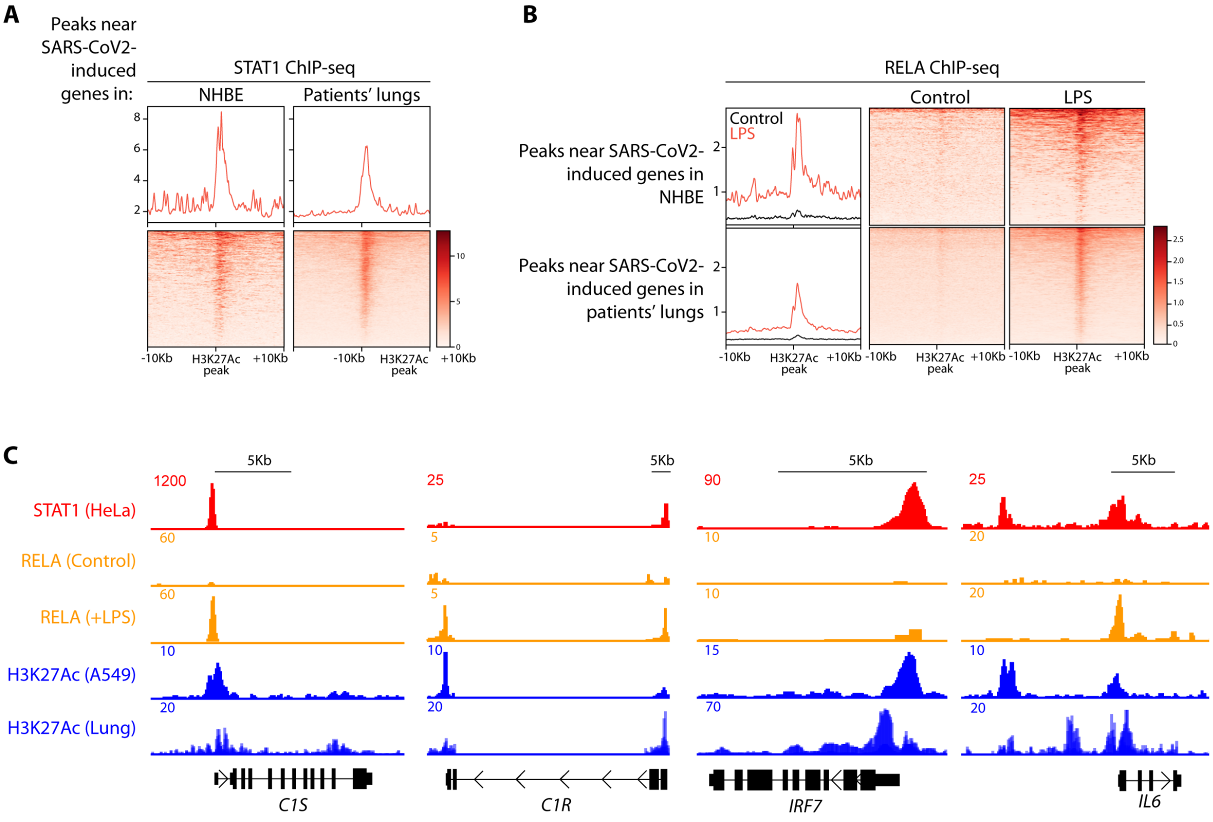

Fig. S3. STAT1 and RELA bind SARS-CoV2-induced genes. (**A-B**) Heatmap showing STAT1 (**A**) and RELA (**B**) ChIP-seq peaks centered at H3K27 acetylated regions of genes induced by SARS-CoV2 infection in primary human bronchial epithelial (NHBE) cells and lungs of COVID-19 patients. In **B**, tracks show RELA profiles before (control) and after (LPS) treatment with LPS. (**C**) STAT1, RELA and H3K27Ac ChIP-seq tracks showing the *C1S*, *C1R*, *IRF7* and *IL6* gene loci. Data are from GSE147507, ENCODE (H3K27Ac and STAT1 ChIP-seqs) and from GSE132018 (RELA).

Fig S4

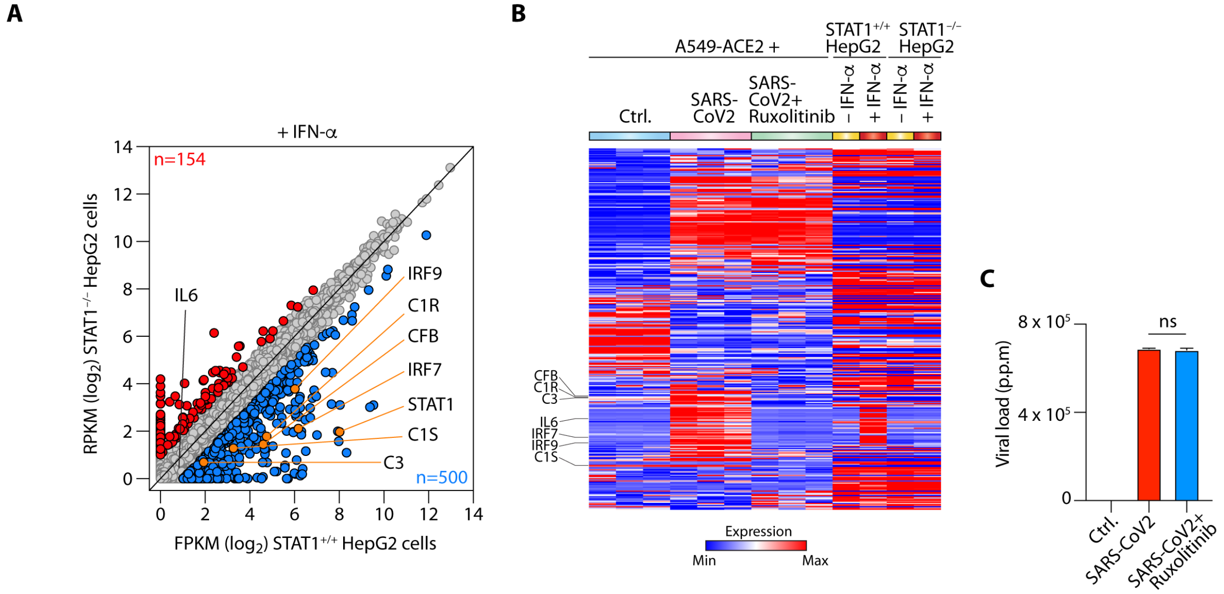

Fig. S4. STAT1 dependence of SARS-CoV2-induced genes. (**A**) Scatter plot comparing the expression of all genes between STAT1 wild-type (*STAT1*$^{+/+}$) and STAT1 knockout (*STAT1*$^{-/-}$) HepG2 cells after interferon (IFN)-α treatment. Differentially expressed genes (Fold change>2) are highlighted in blue (down-regulated in knockout) and red (up-regulated in knockout) and selected key complement and interferon pathway genes highlighted in orange. *IL6* is also marked but not significantly expressed or changed. (**B**) Heatmap showing expression of genes in **Fig. 4C** comparing *STAT1*$^{-/-}$ versus *STAT1*$^{+/+}$ wildtype HepG2 cells. For better visualization, **Fig. 4C** is replicated on the left of this heatmap. Genes are kept in the same order as **Fig. 4C**. Highlighted are selected key complement and interferon pathway genes on the left. Data in **A-B** are from GSE98372. (**C**) Viral load in A549-ACE2 cells transduced with SARS-CoV2 with and without Ruxolitinib (please see also **Fig 4C**). ns, not significant.

Supplementary Table legends

Table S1. Normalized expression of all transcripts in GSE147507 and SRP257667 and GSEA outputs used in Figure 1. (**A**) Normalized expression (TPM) of all transcripts in GSE147507. (**B**) Enriched genesets (FDR q-value <0.25) from GSEA comparisons in **Fig. 1**. (**C**) Normalized expression of all transcripts from ferrets infected with Mock or SARS-CoV2 (GSE147507). (**D**) Normalized expression (TPM) of all transcripts across all samples in SRP257667. (**E**) Enriched genesets (FDR q-value <0.25) from GSEA comparisons in **Fig S1**.

Table S2. List of CD46, C3aR and Interferon alpha/beta target genes curated experimentally for CD46 and from literature for the rest.

Table S3. Differentially expressed genes in SARS-CoV2-infected cells. (**A**) Differentially expressed genes (FC ± 1.5 at FDR <0.05) in NHBE and A549 cells infected with SARS-CoV2 versus control virus. (**B**) STAT1 and RELA bound regions in genes differently regulated by SARS-CoV2 in NHBE cells.

Table S4. GSEA-based drug prediction. (**A**) GSEA predicted drugs used in **Fig. 4**. (**B**) Genes induced/repressed by SARS-CoV2 in A549 cells that are affected or unaffected by Ruxolitinib (data from GSE147507). (**C**) Expression of all transcripts in STAT1 wildtype versus STAT1 knockout HepG2 cells. Data are from GSE98372. (**D**) Genes induced/repressed by Ruxolitinib in MCF7 cells (data from GSE131300) or by systemic Baricitinib treatment in mice (data from GSE61555).
